# A New Method to Detect Variants of SARS-CoV-2 Using Reverse Transcription Loop-Mediated Isothermal Amplification Combined with a Bioluminescent Assay in Real Time (RT-LAMP-BART)

**DOI:** 10.3390/ijms241310698

**Published:** 2023-06-27

**Authors:** Takahiro Iijima, Jun Sakai, Dai Kanamori, Shinnosuke Ando, Tsutomu Nomura, Laurence Tisi, Paul E. Kilgore, Neil Percy, Hikaru Kohase, Satoshi Hayakawa, Shigefumi Maesaki, Tomonori Hoshino, Mitsuko Seki

**Affiliations:** 1 Division of Pediatric Dentistry, Department of Human Development and Fostering, Meikai University School of Dentistry, Sakado 350-0283, Japan; 2 Department of Infectious Disease and Infection Control, Saitama Medical University, Moroyama 350-8550, Japan; 3 Division of Dental Anesthesiology, Department of Diagnostic and Therapeutic Sciences, Meikai University School of Dentistry, Sakado 350-0248, Japan; 4 Division of Otolaryngology, Department of Comprehensive Medical Sciences, Meikai University School of Dentistry, Sakado 350-0248, Japan; 5 Erba Molecular–Lumora, Ely CB7 4EA, UK; 6 Department of Pharmacy Practice, Eugene Applebaum College of Pharmacy & Health Sciences, Wayne State University, Detroit, MI 48201, USA; 7 3M Company, St. Paul, MN 55144, USA; 8 Division of Microbiology, Department of Pathology and Microbiology, Nihon University School of Medicine, Tokyo 113-8602, Japan

**Keywords:** SARS-CoV-2, variants of concern, spike protein, loop-mediated isothermal amplification

## Abstract

Coronavirus disease 2019 (COVID-19) is caused by severe acute respiratory syndrome coronavirus 2 (SARS-CoV-2), of which there are several variants. The three major variants (Alpha, Delta, and Omicron) carry the N501Y, L452R, and Q493R/Q498R mutations, respectively, in the *S* gene. Control of COVID-19 requires rapid and reliable detection of not only SARS-CoV-2 but also its variants. We previously developed a reverse transcription loop-mediated isothermal amplification assay combined with a bioluminescent assay in real time (RT-LAMP-BART) to detect the L452R mutation in the SARS-CoV-2 spike protein. In this study, we established LAMP primers and peptide nucleic acid probes to detect N501Y and Q493R/Q498R. The LAMP primer sets and PNA probes were designed for the N501Y and Q493R/Q498R mutations on the *S* gene of SARS-CoV-2. The specificities of RT-LAMP-BART assays were evaluated using five viral and four bacterial reference strains. The sensitivities of RT-LAMP-BART assays were evaluated using synthetic RNAs that included the target sequences, together with RNA-spiked clinical nasopharyngeal and salivary specimens. The results were compared with those of conventional real-time reverse transcription-polymerase chain reaction (RT-PCR) methods. The method correctly identified N501Y and Q493R/Q498R. Within 30 min, the RT-LAMP-BART assays detected up to 100–200 copies of the target genes; conventional real-time RT-PCR required 130 min and detected up to 500–3000 copies. Surprisingly, the real-time RT-PCR for N501Y did not detect the BA.1 and BA.2 variants (Omicron) that exhibited the N501Y mutation. The novel RT-LAMP-BART assay is highly specific and more sensitive than conventional real-time RT-PCR. The new assay is simple, inexpensive, and rapid; thus, it can be useful in efforts to identify SARS-CoV-2 variants of concern.

## 1. Introduction

Coronavirus disease 2019 (COVID-19), caused by SARS-CoV-2, has spread worldwide [1]; some variants show increased infectivity, morbidity, and mortality [2]. Three variants of concern (VOCs) (Alpha, Delta, and Omicron) bear the N501Y, L452R, and Q493R/Q498R mutations, respectively, in the *S* gene [3].

Rapid and reliable detection of SARS-CoV-2 variants is essential. However, conventional and real-time reverse transcription-polymerase chain reaction (RT-PCR) methods are time-consuming (>2 h), complex, and require expensive equipment. Moreover, some mutations, e.g., N501Y, are challenging to detect with real-time RT-PCR, as also noted by commercial suppliers of widely used kits [4]. This situation creates a void in our abilities to follow viral spread and calls for developing assays with greater flexibility in detecting mutant viruses.

The reverse transcription loop-mediated isothermal amplification (RT-LAMP) assay combined with a bioluminescent assay in real-time (BART) method (RT-LAMP-BART) is a simple and rapid (≤1 h) alternative to conventional and real-time RT-PCR [5]. RT-LAMP has been used to detect the coronaviruses that cause SARS and Middle East Respiratory Syndrome (MERS) [6,7]. Generally, real-time monitoring using molecular assays such as RT-PCR uses fluorescence to detect amplicons. Such tests can only be performed in well-equipped laboratories. Amplification during the RT-LAMP assay is monitored via the precipitation of magnesium pyrophosphate by newly synthesized DNA; this approach is more straightforward than fluorescence monitoring. BART is a real-time, closed-tube luminescence reporting system that enables continuous monitoring of amplification progress using simple equipment [8]. BART uses bioluminescence to assess the exponential increase in inorganic pyrophosphate produced during isothermal amplification of a specific nucleic acid target. BART requires only a simple light detector. Thus, combining RT-LAMP with BART overcomes the limitations of conventional and real-time RT-PCR and is appropriate for use in resource-limited laboratories.

Previously, we used the RT-LAMP-BART assay to detect SARS-CoV-2 containing the L452R mutation in the spike protein [9]. Here, we established LAMP primers and peptide nucleic acid (PNA) probes to detect N501Y and Q493R/Q498R, then explored the analytical specificity and sensitivity of the RT-LAMP-BART assay. Our findings point to PNA-based mutation detection as an important novel tool for monitoring rapidly evolving viruses such as SARS-CoV-2.

## 2. Results

### 2.1. Analytical Reactivity and Specificity

**Figure 1:**
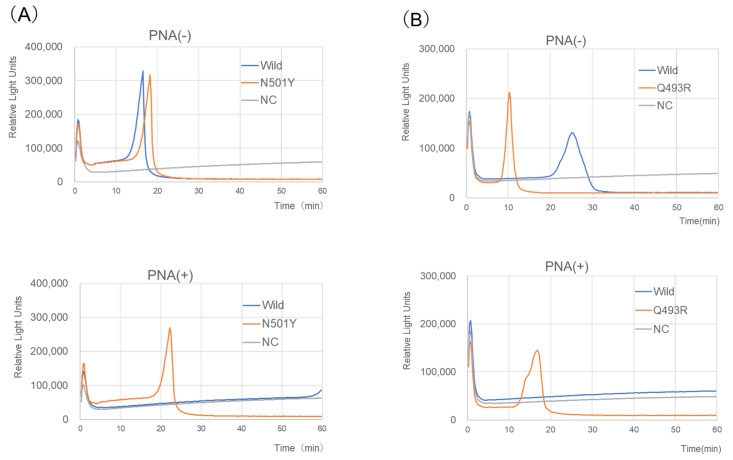
N501Y-RT-LAMP-BART (**A**) and Q493R-RT-LAMP-BART (**B**) assay data with PNA and without PNA derived via real-time monitoring of the lights on the tube using the 3M™ Molecular Detection Instrument (MDS100, 3M, USA). Assayed samples were synthetic *S* gene RNA of SARS-CoV-2 with a point mutation, N501Y (A1501U) for N501Y-RT-LAMP-BART, and Q493R (A1478G) and Q498R (A1493G) for Q493R-RT-LAMP-BART (positive control, 5 × 10^4^ RNA copies), SARS-CoV-2 RNA (wild-type, JPN/AI/1-004, 5 × 10^6^ RNA copies), and DW.

Amplification during the RT-LAMP-BART assay was confirmed by real-time monitoring of light output. Upon addition of a PNA probe, the assay revealed mutations (N501Y or Q493R/Q498R) in the SARS-CoV-2 *S* gene (Figure 1). Briefly, 5 × 10^4^ copies of the N501Y and Q493R/Q498R genes were detected in approximately 20 min, whereas 5 × 10^6^ copies of the wild-type gene yielded no signal over 60 min (Figure 1). The products were directly sequenced and compared with the targeted *S* gene regions F1 to B1c (Supplementary Figure S1; bases 1465–1523, N501Y-RT-LAMP; bases 1452–1507, Q493R-RT-LAMP). The sequences were consistent with expectations (Supplementary Figure S2), indicating that both assays were highly specific.

Both assays identified the mutations with no false positives (Table 1). Six strains with N501Y [B.1.1.7 (Alpha), B.1.351 (Beta), P.1 (Gamma), BA.1, and two BA.2 (Omicron)] were detected by the N501Y-RT-LAMP-BART assay; three other SARS-CoV-2 strains with N501 [two wild-type strains and B.1.617.2 (Delta)] and the other respiratory pathogens were not detected (Table 1). As previously reported [9], only B.1.617.2 was detected by the L452R-RT-LAMP-BART assay; the other eight SARS-CoV-2 strains with L452 and the other respiratory pathogens were not detected. Three SARS-CoV-2 strains with Q493R and Q498R [BA.1 and two BA.2 (Omicron)] were detected by the Q493R-RT-LAMP-BART assay. Non-Omicron SARS-CoV-2 strains and the other respiratory pathogens were not detected.

We compared the *S* gene sequences of 20 coronavirus strains in the primer regions (Supplementary Figure S3). In silico analysis suggested that the N501Y-RT-LAMP and Q493R-RT-LAMP assays would distinguish N501Y (A1501U) and Q493R (A1478G)/Q498R (A1493G) strains from all coronaviruses lacking the target mutations (Supplementary Figure S3). Thus, combined with the RT-LAMP-BART method of the L452R protocol [9], the new methods detecting N501Y and Q493R/Q498R distinguish SARS-CoV-2 VOCs from other variants.

In terms of real-time PCR, the L452R assay detected only the B.1.617.2 variant, which contained the L452R mutation in the spike protein. The assay for the Omicron mutation in the spike protein (G339D) detected the BA.1 and BA.2 variants; it did not detect other variants or the wild type. Surprisingly, the N501Y assay did not detect the BA.1 and BA.2 Omicron variants that exhibited N501Y. In silico analysis confirmed that the RT-PCR primers and probe for N501Y sufficiently annealed to the N501Y variants B.1.1.7, B.1.351, and P.1, but not BA.1 or BA.2 because of four additional mutations within the target regions of BA.1 and BA.2 (Q493R, A1478G; G496S, G1486A; Q498R, A1493G; and Y505H, T1513C). However, it did detect other N501Y variants (B.1.1.7, B.1.351, and P.1). No assay detected wild-type SARS-CoV-2 or other respiratory pathogens.

### 2.2. Reaction Time and Analytical Sensitivity

The reaction times and analytical sensitivities of all assays are shown in Table 2. The new method detected mutations within 30 min; conventional real-time RT-PCR required 105–130 min. LAMP products were measured in real-time according to light output. The RT-LAMP-BART assays were more sensitive than conventional real-time RT-PCR. RT-LAMP-BART detected 100–200 copies/sample; in contrast, conventional real-time RT-PCR for N501Y, L452R, and G339D detected up to 3000, 3000, and 500 copies/sample, respectively (Table 2).

### 2.3. RT-LAMP-BART Assays of RNA-Spiked Specimens

For RNA-spiked NPS specimens, the N501Y-, L452R-, and Q493R-RT-LAMP assays detected 500 copies/sample; thus, the sensitivities were slightly reduced. For RNA-spiked SAL specimens, the analytical sensitivities of the three RT-LAMP assays decreased from 100–200 copies to 5000 copies/sample (Table 2). On the RT-LAMP-BART assay, reaction condition 60 °C was the best temperature, and these results were the same even if the reaction time was increased to 60 min. For RNA-spiked NPS specimens, the conventional real-time RT-PCR assays for N501Y, L452R, and G339D exhibited slightly attenuated sensitivities (5000 copies/sample). For RNA-spiked SAL specimens, the sensitivities decreased to 50,000 copies/sample (Table 2).

## 3. Discussion

The RT-LAMP assay allows simple and rapid detection of SARS-CoV-2 genes [10]. RT-LAMP tests for SARS-CoV-2 have been developed and clinically validated worldwide; the necessary time is 20–60 min, and the limit of detection is 5–500 copies of viral RNA [2,11,12,13,14]. RT-LAMP facilitates point-of-care testing without the requirement for specific equipment; this capability is important in remote regions or when screening large populations. Clinics in pandemic epicenters require high-throughput capacity, sample handling automation, sensitive assay performance, and rapid results. The sensitivity and specificity of the RT-LAMP assays were reported at 87.0–100% and 97.6–100%, which do not differ from the parameters of real-time RT-PCR [2,11,12,13,14]. We now show RT-LAMP, combined with PNA probes, is also an efficient tool for mutation detection in SARS-CoV-2.

To facilitate more rapid detection, we added BART to RT-LAMP. BART continuously monitors the enzymatic conversion of a byproduct (pyrophosphate, PPi) of nucleic acid amplification to adenosine triphosphate (ATP) via bioluminescence generated by firefly luciferase [8]. The assay exhibits a unique light peak, and the timing of the peak depends on the concentration of target nucleic acid; positive/negative scoring is possible. The time to the peak is short, results are rapid, and light detection is simple. The only hardware required is an isothermal heating block and a photodiode-based light detection system. No light source irradiates samples, no expensive filters are necessary, and there is no thermocycling. All consumables are off-the-shelf; the cost is identical to that of fluorescence reporter systems. There is no need for an expensive fluorescence/quenching probe.

For simple and rapid extraction of viral RNA from biological samples, Loopamp™ Viral RNA Extraction Reagent (Eiken Chemical Co., Ltd.) was used in the RNA-spiked clinical specimen experiment in this study. This technique does not need a centrifuge, and with just gently dissolving a cotton swab or saliva into the extraction reagent at room temperature, the virus contained in the sample can be solubilized and the RNA can be released into the solution.

We previously used the RT-LAMP-BART assay to detect SARS-CoV-2; the detection limit was approximately 80 copies. Fei et al. (2021) enhanced the sensitivity to 10 copies using a digital PCR approach [15]. Here, we detected *S* gene point mutations. Within 30 min, RT-LAMP-BART detected 100–200 purified RNA molecules; conventional real-time RT-PCR required 500–3000 copies to yield a positive signal in 130 min. Thus, RT-LAMP-BART is more rapid and sensitive.

RT-LAMP-BART reactions were inhibited by substances in RNA-spiked samples, similar to conventional real-time RT-PCR reactions. Previously, we did not observe such interference with LAMP reactions [16,17,18]. Gandelman et al., reported that LAMP-BART was robust, reliable when using urine specimens, and unaffected by rapid DNA preparation [8]. As previously mentioned [9], the inhibition of RT-LAMP-BART assays by biological substances is weaker than that of conventional real-time RT-PCR assays.

We also showed that RT-LAMP-BART can detect SARS-CoV-2 VOCs. Surprisingly, we found that real-time RT-PCR for N501Y did not detect the BA.1 and BA.2 variants (Omicron) that exhibited the N501Y mutation. However, it detected other variants with that mutation (B.1.1.7, B.1.351, and P.1). As described in the accompanying instructions, the commercial detection kit for the N501Y mutation using real-time RT-PCR technology is also unsuited for the detection of Omicron variants [4]. This may be explained by four additional mutations in the primer target region (Q493R, A1478G; G496S, G1486A; Q498R, A1493G; and Y505H, T1513C), which are likely to influence the real-time RT-PCR reaction.

PNA, a synthetic DNA analog, terminates the elongation of oligonucleotide primers by either binding to the template or competing with the primers [19]. When PNA hybridizes to DNA or RNA, other primers and probes cannot anneal, particularly in the 3′ terminal region; this leads to the cessation of nucleic acid extension. PNA does not directly interact with DNA polymerase or the RT reaction. We found that, during RT-LAMP-BART, the PNA probe was able to interrupt the elongation of LAMP primers via robust PNA binding to wild-type RNA but not to variant RNAs. The N501Y and Q493R/Q498R mutations in the spike protein were detected within 20 min, with a sensitivity of approximately 200 copies/reaction. Compared with real-time RT-PCR, the RT-LAMP-BART assay is more robust in the detection of VOCs.

This study had several limitations. To approve the performance of the established assay in real-world settings, additional work using clinical specimens from patients is required. In addition, new arising SARS-CoV-2 variants, such as XBB, BQ.1, etc. [3], should be examined by the established method.

## 4. Conclusions

The RT-LAMP-BART method detected SARS-CoV-2 VOCs; its analytical sensitivity and specificity were better than those parameters for real-time RT-PCR. The RT-LAMP with PNA approach is clearly generic, as it worked on a number of mutations. Our method is helpful in the detection of SARS-CoV-2 VOCs. It is a powerful novel tool for following viral evolution with high sensitivity, even in poorly equipped healthcare facilities such as those in many low-income countries. In future pandemics, this may also enable better tracing of VOCs in these areas.

## 5. Materials and Methods

### 5.1. Viral and Bacterial Strains

We used 18 viral and 4 respiratory bacterial strains to evaluate the RT-LAMP-BART assay. SARS-CoV-2 strains were provided by the National Institute of Infectious Diseases and Nihon University (Tokyo, Japan) (JPN/AI/1-004 and JPN/TY/WK-521); Vircell (Granada, Spain) (SARS-CoV-2 B.1.1.7 [hCoV-19/Spain/AN-HUSC_24581802/2020], B.1.351 [hCoV-19/Spain/GA-CHUVI-19118872/2020], P.1 [hCoV-19/Spain/GA-CHUVI-19250962/2021], and B.1.617.2 [hCoV-19/Spain/GA-CHUVI-33984566/2021]; and Twist Bioscience (San Francisco, CA, USA) (BA.1 [hCoV-19/Hong Kong/HKU-211129-001/2021], BA.2 [hCoV-19/Australia/QLD2568/2021], and BA.2 [hCoV-19/England/MILK-2DF642C/2021]). The other viral species were SARS-CoV (HKU-39849) and MERS-CoV (Betacoronavirus England 1) (Vircell); human coronaviruses OC43 and 229E (American Type Culture Collection, Manassas, VA, USA); influenza A (H1N1) (PR8 and Pdm2009); and respiratory syncytial virus (Long, subgroups A and B) (Prof. Tetsuo Nakayama, Kitasato University, Sagamihara, Japan). The respiratory bacterial species were *Haemophilus influenzae* (Rd KW20), *Streptococcus pneumoniae* (6305), *Escherichia coli* (DH5α), and *Neisseria meningitidis* (HY0001) (Hanyang University, Ansan, Republic of Korea).

### 5.2. Preparation of Genomic DNA/RNA

Genomic DNAs of *S*. *pneumoniae*, *H*. *influenzae,* and *E*. *coli* were extracted using a QIAamp DNA Mini kit (Qiagen, Valencia, CA, USA) in accordance with the manufacturer’s protocol. The genetic material of the viral species and the *N. meningitidis* strain were transferred as purified RNA/DNA. Genomic DNA was analyzed using a NanoDrop 1000/2000 instrument (Thermo Fisher Scientific Inc., Waltham, MA, USA). The numbers of genome copies were calculated based on a molecular size of 2.04 Mbp for *S*. *pneumoniae* (R6; GenBank accession number, AE007317), 5.2 Mbp for *E*. *coli* (CFT073; GenBank accession number, AE014075.1), and 1.83 Mbp for *H*. *influenzae* (RD KW20; GenBank accession number, NC000907). The copy numbers of genomic RNA in SARS-CoV-2 samples were calculated using real-time RT-PCR [20]. The copy numbers of SARS-CoV, MERS-CoV, human coronavirus, influenza virus, and respiratory syncytial virus (RSV) were determined from the manufacturer’s instructions. Each DNA/RNA sample was adjusted to ≥10^4^ copies/µL, then used to evaluate the specificities of the RT-LAMP-BART and conventional real-time RT-PCR methods [21,22].

### 5.3. Synthetic SARS-CoV-2 RNA and Analytical Sensitivity

When exploring analytical sensitivities, 1500-base-long synthetic SARS-CoV-2 RNAs that included the *S* gene N501Y (A1501U), Q493R (A1478G), and Q498R (A1493G) mutations (Supplementary Figure S4) were obtained from Grainer Japan (Tokyo, Japan). We also used the synthetic RNA of the L452R *S* gene, which was previously described [9]. To explore analytical sensitivities, dilutions (to 5 × 10^4^, 5 × 10^3^, 3 × 10^3^, 500, 200, 100, 50, and 5 copies/sample) were tested in the RT-LAMP-BART and real-time RT-PCR assays; the results were compared. All assays were performed in triplicate.

### 5.4. RNA-Spiked Clinical Specimens

The analytical sensitivity of the LAMP assay was evaluated using RNA-spiked clinical samples, including nasopharyngeal swabs (NPS) and saliva (SAL) specimens, obtained from five healthy volunteers. The NPS specimens were collected using FLOQSwab^TM^ 534CS01-E and Universal Transport Medium (UTM^®^; Copan, Brescia, Italy) and stored at –20 °C. SAL specimens were collected in sterilized tubes and frozen at –20 °C. NPS and SAL samples were subjected to Loopamp™ Virus RNA extraction reagent (Eiken Chemical Co., Tokyo, Japan) according to the manufacturer’s instructions. Synthetic SARS-CoV-2 RNAs were spiked into the specimens and used for evaluation. The dilutions (5 × 10^4^, 5 × 10^3^, 3 × 10^3^, 500, 200, 100, 50, and 5 copies per reaction) were used for the RT-LAMP-BART and real-time RT-PCR assays. The results were compared between the RT-LAMP-BART and conventional real-time RT-PCR methods. Each reaction was performed in triplicate.

### 5.5. LAMP Primer Design

Primer Explorer version 5 software [23] was used to define two LAMP primer sets (GISAID ID: EPI_ISL_906081 for *S* gene N501Y [A1501U] and EPI_ISL_6841980 (Omicron BA.1) for Q493R/Q498R [A1478G/A1493G]; Supplementary Figure S1). Both primer sets included outer primers (F3 and B3), inner primers (FIP and BIP), and loop primers (LF and LB) (Table 3). Two PNA probes for wild-type sequences (N501 [A1501] and Q493 [A1478] plus Q498 [A1493]) were also developed (Table 3, Supplementary Figure S1). The PNA probes were complementary to the wild-type sequence; they interfered with the RT-LAMP reaction by binding to the wild-type sequence, thereby prohibiting LAMP amplification.

### 5.6. RT-LAMP-BART Assay

Lyophilized BART master reagent (Erba Mannheim, Ely, UK) was dissolved to prepare 1.6 μM solutions of FIP and BIP, 0.4 μM solutions of F3 and B3, 0.8 μM solutions of LF and LB, a 0.1 μM solution of PNA, and 2 × 125 mM bicine acetate buffer with 2 mM MgCl_2_. We used three LAMP primer plus PNA probe sets: N501Y-RT-LAMP, L452R-RT-LAMP [9], and Q493R-RT-LAMP (Table 3). The final volume of each sample was adjusted with medical-grade water to 15 μL to which 5 μL of template DNA/RNA was added (20 μL total volume). The reaction mixtures were covered with mineral oil and incubated at 60 °C for 60 min; during incubation, light output from each tube as a function of time was measured using a real-time LAMP-BART apparatus (PCRuN, Biogal Galed Lab, Kibbutz Galed, Israel, or 3M™ Molecular Detection Instrument, MDS100, 3M, St. Paul, MN, USA). After an initial decrease in light emission during the first few minutes, the sample was considered positive if the light signal exhibited a substantial increase followed by a decrease [8]. Peak light output was identified in accordance with the manufacturer’s protocol.

### 5.7. Analysis of RT-LAMP-BART Products

Amplified LAMP products were sequenced by Sigma-Aldrich Japan, Tokyo, Japan using the following F2 primers for N501Y-RT-LAMP and Q493R-RT-LAMP: N501Y-F2, 5′-CTG AAA TCT ATC AGG CCG GTA G-3′; and Q493R-F2, 5′-TTT CAA CTG AAA TCT ATC AGG CC-3′.

### 5.8. In Silico Analysis

We aligned the primer *S* gene sequences on the sequences with 20 coronavirus strains: 14 SARS-CoV-2 strains [GenBank accession number and GISAID ID: NC_045512.2 (Wild), EPI_ISL_601443 (Alpha), EPI_ISL_660190 (Beta), EPI_ISL_906081 (Gamma), EPI_ISL_1419152 (Delta), EPI_ISL_8750789 (Epsilon), EPI_ISL_8005633 (Zeta), EPI_ISL_1083809 (Eta), EPI_ISL_8158591 (Iota), EPI_ISL_8259884 (Mu), EPI_ISL_6841980 (Omicron BA.1), EPI_ISL_9097416 (Omicron BA.2), EPI_ISL_13241867 (Omicron BA.4), EPI_ISL_12703378 (Omicron BA.5)], SARS-CoV-1 (NC_004718.3), MERS-CoV (NC_019843.3)], and four human coronaviruses [α-coronaviruses AF304460.1 (229E) and AY567487.2 (NL63), and β-coronaviruses AY391777.1 (OC43) and NC_006577.2 (HKU1)] (Supplementary Figure S3). We used in silico analysis (FastPCR [24]) to explore whether the two RT-LAMP assays distinguished the target mutations.

### 5.9. Real-Time RT-PCR

Conventional real-time RT-PCR (using TaqMan probes) assays to detect N501Y, L452R [21], and G339D (Omicron) [22] were performed using the One-Step Prime Script RT-PCR kit (TaKaRa Bio, Kusatsu, Japan); the primers and probe are shown in Supplementary Table S1. The cycling conditions for N501Y [21], L452R [21], and G339D [22] were conditions previously reported for the LightCycler480 system (Roche Diagnostics, Basel, Switzerland).

### 5.10. Ethics Statement

We collected NPS and SAL specimens from five healthy volunteers at the Meikai University School of Dentistry. The study protocol was reviewed and approved by the Institutional Review Board of the Meikai University School of Dentistry (IRB #A2001). Written informed consent was obtained from the five volunteers.

## Figures and Tables

**Table 1:** Comparison of RT-LAMP-BART and real-time PCR assays detecting N501Y, L452R, and Omicron’s spike mutations (Q493R/G339D) of SARS-CoV-2.

Virus/Bacteria	Results of Two Assays					
	RT-LAMP-BART			Real-Time PCR		
	N501Y	L452R	Q493R	N501Y	L452R	G339D
SARS-CoV-2						
JPN/AI/1-004 ^b^	− ^a^	−	−	−	−	−
JPN/TY/WK-521 ^b^	−	−	−	−	−	−
B.1.1.7 (ALPHA) hCoV-19/Spain/AN-HUSC_24581802/2020 ^c^	+	−	−	+	−	−
B.1.351 (BETA) hCoV-19/Spain/GA-CHUVI-19118872/2020 ^c^	+	−	−	+	−	−
P.1 (GAMMA) hCoV-19/Spain/GA-CHUVI-19250962/2021 ^c^	+	−	−	+	−	−
B.1.617.2 (DELTA) hCoV-19/Spain/GA-CHUVI-33984566/2021 ^c^	−	+	−	−	+	−
BA.1 (Omicron) hCoV-19/Hong Kong/HKU-211129-001/2021 ^d^	+	−	+	−	−	+
BA.2 (Omicron) hCoV-19/Australia/QLD2568/2021 ^d^	+	−	+	−	−	+
BA.2 (Omicron) hCoV-19/England/MILK-2DF642C/2021 ^d^	+	−	+	−	−	+
SARS-CoV ^c^	−	−	−	−	−	−
MERS-CoV Betacoronavirus England 1 ^c^	−	−	−	−	−	−
Human CoV OC43 ^e^	−	−	−	−	−	−
Human CoV 229E ^e^	−	−	−	−	−	−
Influenza A (H1N1) PR8 ^f^	−	−	−	−	−	−
Influenza A (H1N1) Pdm2009 ^f^	−	−	−	−	−	−
RSV Long ^f^	−	−	−	−	−	−
RSV subgroups A ^f^	−	−	−	−	−	−
RSV subgroups B ^f^	−	−	−	−	−	−
*Haemophilus influenzae* Rd KW20	−	−	−	−	−	−
*Streptococcus pneumoniae* 6305	−	−	−	−	−	−
*Neisseria meningitidis* HY0001 ^g^	−	−	−	−	−	−
*Escherichia coli* DH5a	−	−	−	−	−	−

^a^ +, positive; −, negative; Source: ^b^ National Institute of Infectious Diseases; ^c^ Vircell; ^d^ Twist Bioscience; ^e^ ATCC; ^f^ Kitasato University; ^g^ Hanyang University.

**Table 2:** Analytical sensitivity and reaction time of the RT-LAMP-BART and real-time RT-PCR assays used to detect synthetic SARS-CoV-2 RNA, including the target regions of the *S* gene and the synthetic RNA-spiked nasopharyngeal and saliva specimens.

	Analytical Sensitivity	
	RT-LAMP-BART (Reaction Time, 30 min)	Real-Time RT-PCR (Reaction Time, 105–130 min)
Purified RNA		
N501Y	200 copies/reaction ^a^	3000
L452R	100	3000
Omicron: Q493R, LAMP; G339D, PCR	100	500
RNA-spiked specimens		
Nasopharyngeal swab ^b^		
N501Y	500	5000
L452R	500	5000
Omicron: Q493R, LAMP; G339D, PCR	500	5000
saliva ^b^		
N501Y	5000	50,000
L452R	5000	50,000
Omicron: Q493R, LAMP; G339D, PCR	5000	50,000

^a^ amount of RNA copies per reaction; ^b^ samples prepared via Loopamp^TM^ Virus RNA extraction reagent (Eiken Chemical Co., Ltd, Tokyo, Japan).

**Table 3:** LAMP primer sets and probes used in this study.

Name	Sequence (5′ to 3′)
N501Y-RT-LAMP	
N501Y_F3	CTA ATC TCA AAC CTT TTG AGA GAG A
N501Y_B3	AAT TAG TAG ACT TTT TAG GTC CAC AA
N501Y_FIP	GGA AAC CAT ATG ATT GTA AAG GAA AGT ACT GAA ATC TAT CAG GCC GGT AG
N501Y_BIP	TAT GGT GT GGT TAC CAA CCA TAA CAG TTG CTG GTG CAT GT
N501Y_LF	ACC TTT AAC ACC ATT ACA AGG TGT
N501Y_LB	AGA GTA GTA GTA CTT TCT TTT GAA CTT CT
N501Y_PNA probe	CCA ACA CCA TT ^a^ A GTG GG
L452R-RT-LAMP [9]	
L452R_F3	GCA AAC TGG AAA GAT TGC TGA T
L452R_B3	TTG GAA ACC ATA TGA TTG TAA AGG A
L452R_FIP	CGG TAA TTA TAA TTA CCA CCA ACC TAG ATG ATT TTA CAG GCT GCG T
L452R_BIP	GTT TAG GAA GTC TAA TCT CAA ACC TAA CAC CAT TAC AAG GTG TGC TA
L452R_LF	TCA AGA TTG TTA GAA TTC CAA GCT AT
L452R_LB	AGA GAG ATA TTT CAA CTG AAA TCT ATC AG
L452R_PNA probe	CCT AAA CAA TCT ATA CA ^b^ G G
Q493R-RT-LAMP	
Q493R_F3	CTA ATC TCA AAC CTT TTG AGA GAG A
Q493R_B3	AGACTT TTT AGG TCC ACA AAC AGT
Q493R_FIP	CGT AAA GGA AAG TAA CAA TTA AAA CCT TTT CAA CTG AAA TCT ATC AGG CC
Q493R_BIP	GTT TCC GAC CCA CTT ATG GTG TGT GGT GCA TGT AGA AGT TC
Q493R_LF	GCA ACA CCA TTA CAA GGT
Q493R_LB	TTG GTC ACC AAC CAT ACA GAG
Q493R/Q498R_PNA probe	TT ^c^ G GAA ACC A ^c^ TA TGA TT ^c^G

^a^ PNA probe was designed to be complementary to the wild-type sequence (U1501 of the *S* gene) and positioned to interfere with the RT-LAMP reaction. ^b^ PNA probe was designed to be complementary to the wild-type sequence (U1355 of the *S* gene) and positioned to interfere with the RT-LAMP reaction. ^c^ PNA probe was designed to be complementary to the wild-type sequence (U1478, U1486, and U1493 of the *S* gene) and positioned to interfere with the RT-LAMP reaction.

## Data Availability

Data is contained within the article or Supplementary Materials.

## Supplementary Materials

The following supporting information can be downloaded at: https://www.mdpi.com/article/10.3390/ijms241310698/s1.
