# Supplementary material for: A New Method to Detect Variants of SARS-CoV-2 Using Reverse Transcription Loop-Mediated Isothermal Amplification Combined with a Bioluminescent Assay in Real Time (RT-LAMP-BART)
Source: Int J Mol Sci. 2023 Jun 27;24(13):10698. doi: 10.3390/ijms241310698 (PMC10341676; doi:10.3390/ijms241310698)
Supplement: Supplementary file 1 [file ijms-24-10698-s001.zip › ijms-2417473-supplementary.pdf]

Supplementary Figure 1

A), Nucleotide sequence of the SARS-CoV-2 *S* gene used to design the N501Y-RT-LAMP-BART primers; B), Nucleotide sequence of the SARS-CoV-2 *S* gene used to design the Q493R-RT-LAMP-BART primers.

The sequences used for the RT-LAMP primers are indicated by arrows.

A)

```
NO          1371
Sequence    GAAGTCTAAT CTCAAACCTT TTGAGAGAGA TATTTCAACT GAAATCTATC
                      |----------------------F3---------------------->             |-----------F2--------

NO          1421
Sequence    AGGCCGGTAG CACACCTTGT AATGGTGTTA AAGGTTTTAA TTGTTACTTT
            -------------------->   <---------------------LF----------------------|             |----------

NO          1471                                  <----------------PNA-----------|
Sequence    CCTTTACAAT CATATGGTTT CCAACCCACT TATGGTGTTG GTTACCAACC
            -----------------F1c------------------->          <-------------------------B1c----------

NO          1521
Sequence    ATACAGAGTA GTAGTACTTT CTTTTGAACT TCTACATGCA CCAGCAACTG
           -----|   |------------------------LB---------------------------> <----------------B2--------------

NO          1571
Sequence    TTTGTGGACC TAAAAAGTCT ACTAATTTGG TTAAAAACAA ATGTGTCAAT
          --| <------------------------B3----------------------|
```

Red text: 1450, G1450A (E484K); 1501, A1501U (N501Y)

B)

```
NO          1371
Sequence    GAAGTCTAAT CTCAAACCTT TTGAGAGAGA TATTTCAACT GAAATCTATC
               |-----------------------F3-------------------------->    |----------------F2-----------------

NO          1421
Sequence    AGGCCGGTAG CACACCTTGT AATGGTGTTG CAGGTTTTAA TTGTTACTTT
           ---------->                      <--------------LF------------------| |---------------F1c----------------

NO          1471      <----------------PNA--------------|
Sequence    CCTTTACGAT CATATAGTTT CCGACCCACT TATGGTGTTG GTCACCAACC
           --------------->                   <--------------------B1c-----------------| |------------LB------------

NO          1521
Sequence    ATACAGAGTA GTAGTACTTT CTTTTGAACT TCTACATGCA CCAGCAACTG
           ---------------->                                     <--------------------B2-----------------| <-------

NO          1571
Sequence    TTTGTGGACC TAAAAAGTCT ACTAATTTGG TTAAAAACAA ATGTGTCAAT
           -----------------B3---------------------|
```

Red text: 1451, A1451C (E484A); 1478, A1478G (Q493R); 1486, G1486A (G496S); 1493, A1493G (Q498R); 1501, A1501T (N501Y); 1513, T1513C (Y505H)

Supplementary Figure 2.
Sequence data of amplified products of (A) N501Y-RT-LAMP-BART and (B) Q493R-RT-LAMP-BART assays.

(A)
TACTTTCCTTTACAATCATATGGTTTCCAACCCACTTATGGTGTTGGTTACCAACCATA

Red text, N501Y(A1501T)

(B)
AGGTTTTAATTGTTACTTTCCTTTACGATCATATAGTTTCCGACCCACTTATGGTG

Red text, Q493R(A1478G)
Red text, G496S(G1486A)
Red text, Q498R(A1493G)
Red text, N501Y(A1501T)

Supplementary Figure 3. The *S* gene sequences of 20 coronavirus strains in the primer/probe regions of RT-LAMP-BART assays. (A), L452R; (B), N501Y; (C), Q493R.

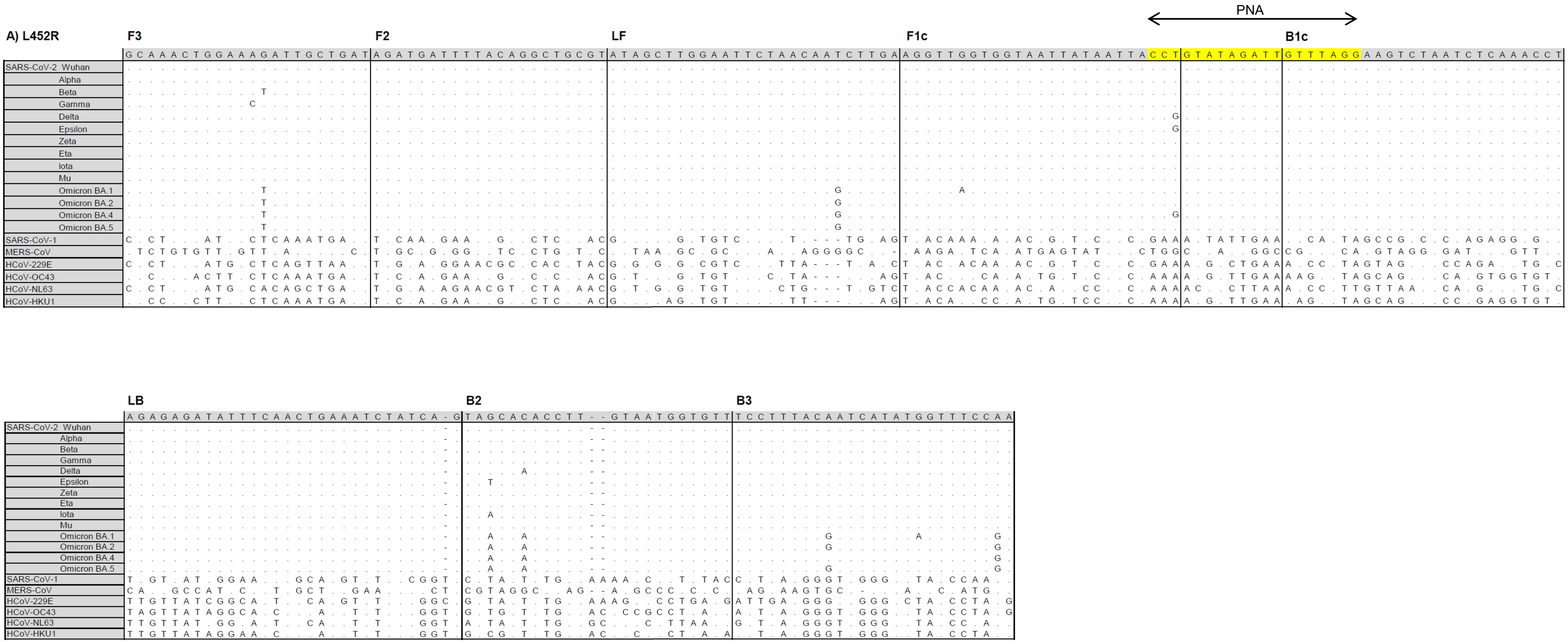

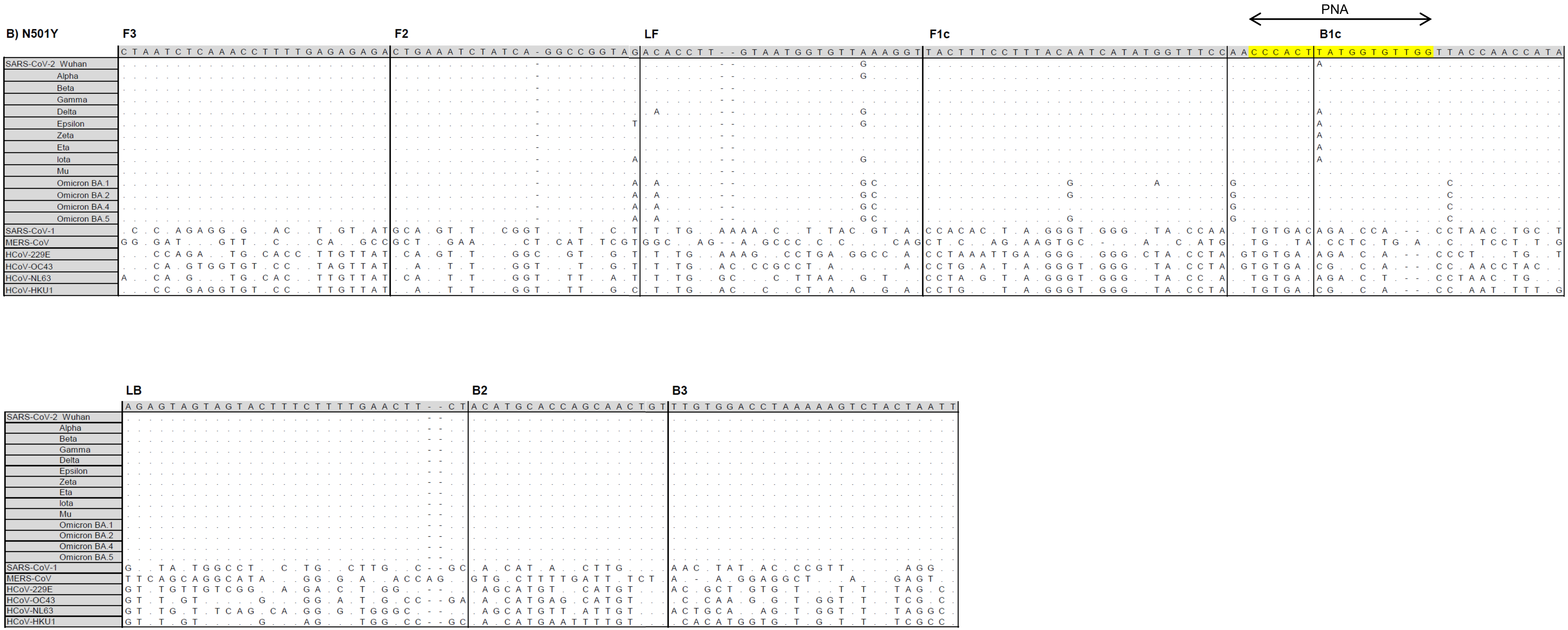
B) N501Y
PNA
F3
F2
LF
F1c
B1c
LB
B2
B3
SARS-CoV-2 Wuhan
Alpha
Beta
Gamma
Delta
Epsilon
Zeta
Eta
Iota
Mu
Omicron BA.1
Omicron BA.2
Omicron BA.4
Omicron BA.5
SARS-CoV-1
MERS-CoV
HCoV-229E
HCoV-OC43
HCoV-NL63
HCoV-HKU1

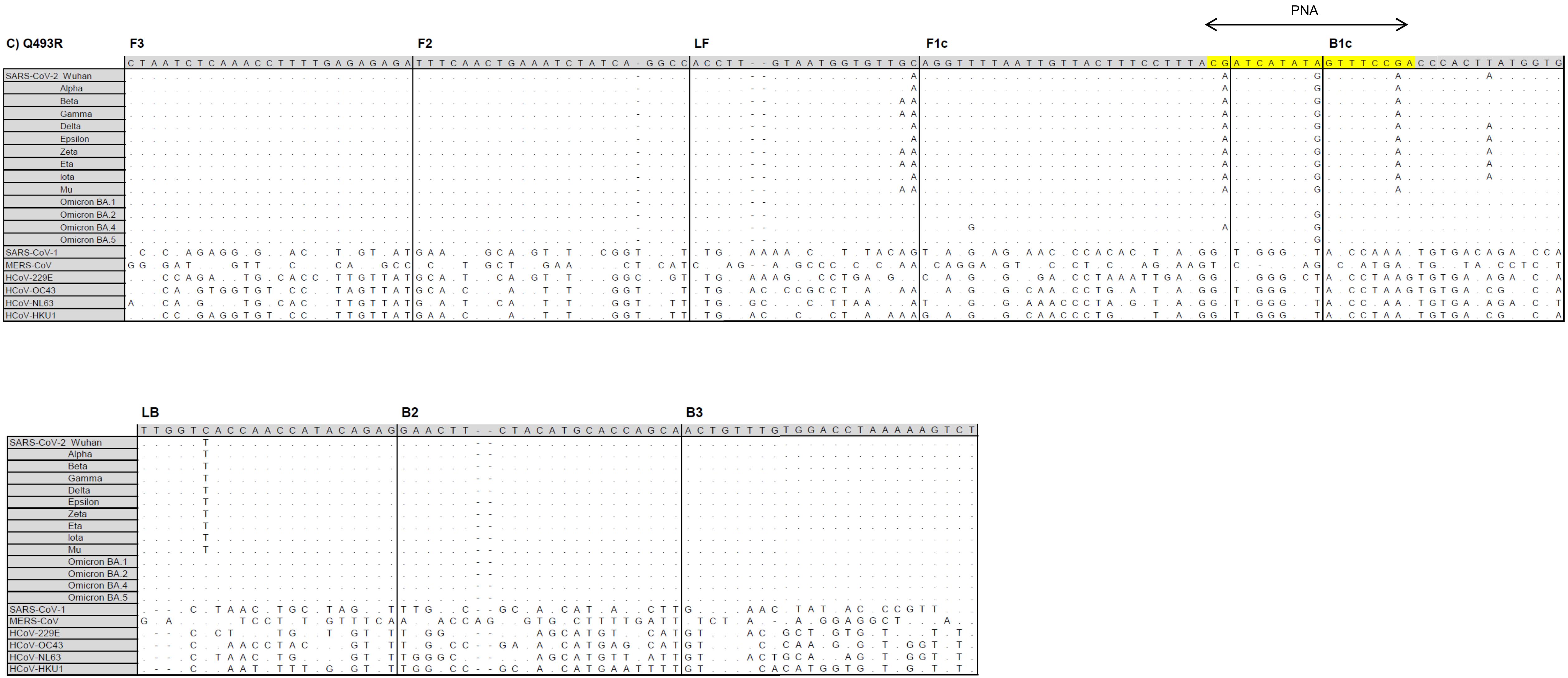

C) Q493R
PNA
F3
F2
LF
F1c
B1c
CTAATCTCAAACCTTTTGAGAGAGA
TTTCAACTGAAATCTATCA-GGCC
ACCTT--GTAATGGTGTTGC
AGGTTTTAATTGTTACTTTCCTTTACG
ATCATATA
GTTTCCGA
CCCACTTATGGTG
LB
B2
B3
TTGGTCACCAACCATACAGAG
GAACTT--CTACATGCACCAGCA
ACTGTTTGTGGACCTAAAAAGTCT
SARS-CoV-2 Wuhan
Alpha
Beta
Gamma
Delta
Epsilon
Zeta
Eta
Iota
Mu
Omicron BA.1
Omicron BA.2
Omicron BA.4
Omicron BA.5
SARS-CoV-1
MERS-CoV
HCoV-229E
HCoV-OC43
HCoV-NL63
HCoV-HKU1

Supplementary Figure 4.
A), Synthetic SARS-CoV-2 RNA including the target region of *S* gene used to design the RT-LAMP assay for N501Y; B), Synthetic SARS-CoV-2 RNA including the target region of *S* gene used to design the RT-LAMP assay for Q493R/Q498R.

A)
gggAGAAACAAAGUGUACGUUGAAAUCCUUCACUGUAGAAAAAGGAAUCUAUCAAACUUCUAACUUUAGAGUCCAACCAACAGA
AUCUAUUGUUAGAUUUCCUAAUAUUACAAACUUGUGCCCUUUUGGUGAAGUUUUUAACGCCACCAGAUUUGCAUCUGUUUAU
GCUUGGAACAGGAAGAGAAUCAGCAACUGUGUUGCUGAUUAUUCUGUCCUAUAUAAUUCCGCAUCAUUUUCCACUUUUAAGU
GUUAUGGAGUGUCUCCUACUAAAUUAAAUGAUCUCUGCUUUACUAAUGUCUAUGCAGAUUCAUUUGUAAUUAGAGGUGAUGA
AGUCAGACAAAUCGCUCCAGGGCAAACUGGAAAGAUUGCUGAUUAUAAUUAUAAAUUACCAGAUGAUUUUACAGGCUGCGUU
AUAGCUUGGAAUUCUAACAAUCUUGAUUCUAAGGUUGGUGGUAAUUAUAAUUACCGGUAUAGAUUGUUUAGGAAGUCUAAUC
UCAAACCUUUUGAGAGAGAUAUUUCAACUGAAAUCUAUCAGGCCGGUAGCACACCUUGUAAUGGUGUUCAAGGUUUUAAUUG
UUACUUUCCUUUACAAUCAUAUGGUUUCCAACCCACU**U***AUGGUGUUGGUUACCAACCAUACAGAGUAGUAGUACUUUCUUUU
GAACUUCUACAUGCACCAGCAACUGUUUGUGGACCUAAAAAGUCUACUAAUUUGGUUAAAAACAAAUGUGUCAAUUUCAACUU
CAAUGGUUUAACAGGCACAGGUGUUCUUACUGAGUCUAACAAAAAGUUUCUGCCUUUCCAACAAUUUGGCAGAGACAUUGCU
GACACUACUGAUGCUGUCCGUGAUCCACAGACACUUGAGAUUCUUGACAUUACACCAUGUUCUUUUGGUGGUGUCAGUGUUA
UAACACCAGGAACAAAUACUUCUAACCAGGUUGCUGUUCUUUAUCAGGAUGUUAACUGCACAGAAGUCCCUGUUGCUAUUCA
UGCAGAUCAACUUACUCCUACUUGGCGUGUUUAUUCUACAGGUUCUAAUGUUUUUCAAACACGUGCAGGCUGUUUAAUAGGG
GCUGAACAUGUCAACAACUCAUAUGAGUGUGACAUACCCAUUGGUGCAGGUAUAUGCGCUAGUUAUCAGACUCAGACUAAUU
CUCCUCGGCGGGCACGUAGUGUAGCUAGUCAAUCCAUCAUUGCCUACACUAUGUCACUUGGUGCAGAAAAUUCAGUUGCUUA
CUCUAAUAACUCUAUUGCCAUACCCACAAAUUUUACUAUUAGUGUUACCACAGAAAUUCUACCAGUGUCUAUGACCAAGACAU
CAGUAGAUUGUACAAUGUACAUUUGUGGUGAUUCAACUGAAUGCAGCAAUCUUUUGUUGCAAUAUGGCAGUUUUUGUACACA
AUUAAACCGUGCUUUAACUGGAAUAGCUGUUGAACAAGACAAAAACACCCAAGAAGUUUUUGCACAAGUCAAACAAAUUUACA
AAACACCACCAAUUAAAGAUUU

*, N501Y (A1501U)

B)
TCTAGTGCGAATAATTGCACTTTTGAATATGTCTCTCAGCCTTTTCTTATGGACCTTGAAGGAAAACAGGGTAATTTCAAAAATCTTA
GGGAATTTGTGTTTAAGAATATTGATGGTTATTTTAAAATATATTCTAAGCACACGCCTATTA[1]TAGTGCGTGAGCCAGAAGATCTCCCT
CAGGGTTTTTCGGCTTTAGAACCATTGGTAGATTTGCCAATAGGTATTAACATCACTAGGTTTCAAACTTTACTTGCTTTACATAGAAG
TTATTTGACTCCTGGTGATTCTTCTTCAGGTTGGACAGCTGGTGCTGCAGCTTATTATGTGGGTTATCTTCAACCTAGGACTTTTCTA
TTAAAATATAATGAAAATGGAACCATTACAGATGCTGTAGACTGTGCACTTGACCCTCTCTCAGAAACAAAGTGTACGTTGAAATCCT
TCACTGTAGAAAAAGGAATCTATCAAACTTCTAACTTTAGAGTCCAACCAACAGAATCTATTGTTAGATTTCCTAATATTACAAACTTGT
GCCCTTTTGA[2]TGAAGTTTTTAACGCCACCAGATTTGCATCTGTTTATGCTTGGAACAGGAAGAGAATCAGCAACTGTGTTGCTGAT
TATTCTGTCCTATATAATC[3]T[3]CGCAC[4]CATTTTT[5]CACTTTTAAGTGTTATGGAGTGTCTCCTACTAAATTAAATGATCTCTGCTTTACTA
ATGTCTATGCAGATTCATTTGTAATTAGAGGTGATGAAGTCAGACAAATCGCTCCAGGGCAAACTGGAAAT[6]ATTGCTGATTATAATTA
TAAATTACCAGATGATTTTACAGGCTGCGTTATAGCTTGGAATTCTAACAAG[7]CTTGATTCTAAGGTTA[8]GTGGTAATTATAATTACCTG
TATAGATTGTTTAGGAAGTCTAATCTCAAACCTTTTGAGAGAGATATTTCAACTGAAATCTATCAGGCCGGTAA[9]CAA[10]ACCTTGTAAT
GGTGTTGC[11]AGGTTTTAATTGTTACTTTCCTTTACG[12]ATCATATA[13]GTTTCC**G**[14]ACCCACTT[15]ATGGTGTTGGTC[16]ACCAACCATACA
GAGTAGTAGTACTTTCTTTTGAACTTCTACATGCACCAGCAACTGTTTGTGGACCTAAAAAGTCTACTAATTTGGTTAAAAACAAATG
TGTCAATTTCAACTTCAATGGTTTAAA[17]AGGCACAGGTGTTCTTACTGAGTCTAACAAAAAGTTTCTGCCTTTCCAACAATTTGGCA
GAGACATTGCTGACACTACTGATGCTGTCCGTGATCCACAGACACTTGAGATTCTTGACATTACACCATGTTCTTTTGGTGGTGTCA
GTGTTATAACACCAGGAACAAATACTTCTAACCAGGTTGCTGTTCTTTATCAGGG[18]TGTTAACTGCACAGAAGTCCCTGTTGCTATT
CATGCAGATCAACTTACTCCTACTTGGCGTGTTTATTCTACAGGTTCTAATGTTTTTCAAACACGTGCAGGCTGTTTAATAGGGGCTG
AAT[19]AT

[1], L212I (T630A) [2], G339D (G1016A) [3], S371L (T1111C, C1112T) [4], S373P (T1117C) [5], S375F (C1124T) [6], K417N (G1251T)
[7], N440K (T1320G) [8], G446S (G1336A) [9], S477N (G1430A) [10], T478K (C1433A) [11], E484A (A1451C) [12], Q493R (A1478G)
[13], G496S (G1486A) [14], Q498R (A1493G) [15], N501Y (A1501T) [16], Y505H (T1513C) [17], T547K (C1640A) [18], D614G (A1841G)
[19], H655Y (C1963T)

Supplementary Table 1. Primer and probe for real-time RT-PCR

Name	Sequence(5' to 3')	Concentration (nM)
N501Y [a]		
S-N501Y_F	TGT TAC TTT CCT TTA CAA TCA TAT GGT TTC	900
S-N501Y_R	GAA AGT ACT ACT ACT CTG TAT GGT TGG TAA CC	900
S-501Y_P	FAM-CAA CCC ACT TAT GGT GTT-MGB	200
L452R [a]		
S-L452R_F	TGA TAG ATT TCA GTT GAA ATA TCT CTC TCA	900
S-L452R_R	AAT CTT GAT TCT AAG GTT GGT GGT AAT TAT	900
S-452R_P	HEX-CTA AAC AAT CTA TAC CGG TAA T-MGB	200
G339D [b]		
S-G339D_F	CAA ACT TCT AAC TTT AGA GTC CAA CCA AC	600
S-G339D_R	CTG TTC CAA GCA TAA ACA GAT GC	600
S-339D_P	FAM-CCC TTT TGA TGA AGT TT-MGB	100

[a] Wang et al., 2021 [21]; [b] Takemae et al., 2022 [22].
